# Development of inducer-free expression plasmids based on IPTG-inducible promoters for *Bacillus subtilis*

**DOI:** 10.1186/s12934-017-0747-0

**Published:** 2017-07-25

**Authors:** Dinh Thi Minh Tran, Trang Thi Phuong Phan, Thanh Kieu Huynh, Ngan Thi Kim Dang, Phuong Thi Kim Huynh, Tri Minh Nguyen, Tuom Thi Tinh Truong, Thuoc Linh Tran, Wolfgang Schumann, Hoang Duc Nguyen

**Affiliations:** 1 Center for Bioscience and Biotechnology, VNUHCMC-University of Science, 227 Nguyen Van Cu Dist. 5, Hochiminh, Vietnam; 2 Laboratory of Molecular Biotechnology, VNUHCMC-University of Science, 227 Nguyen Van Cu Dist. 5, Hochiminh, Vietnam; 3 Department of Biology, Hochiminh City University of Education, 280 An Duong Vuong, Disct. 5, Hochiminh City, Vietnam; 4 Institute of Genetics, University of Bayreuth, 95440 Bayreuth, Germany; 5 Department of Microbiology, VNUHCMC-University of Science, 227 Nguyen Van Cu Dist. 5, Hochiminh, Vietnam

**Keywords:** *Bacillus subtilis*, Inducer-free expression vector, IPTG-inducible promoter, P*spac*, P*grac*, P*grac*100

## Abstract

**Background:**

Besides *Escherichia coli*, *Bacillus subtilis* is an important bacterial species for the production of recombinant proteins. Recombinant genes are inserted into shuttle expression vectors which replicate in both *E. coli* and in *B. subtilis*. The ligation products are first transformed into *E. coli* cells, analyzed for correct insertions, and the correct recombinant plasmids are then transformed into *B. subtilis*. A major problem using *E. coli* cells can be the strong basal level of expression of the recombinant protein which may interfere with the stability of the cells. To minimize this problem, we developed strong expression vectors being repressed in *E. coli* and inducer-free in *B. subtilis*.

**Results:**

In general, induction of IPTG-inducible expression vectors is determined by the regulatory *lacI* gene encoding the LacI repressor in combination with the *lacO* operator on the promoter. To investigate the inducer-free properties of the vectors, we constructed inducer-free expression plasmids by removing the *lacI* gene and characterized their properties. First, we examined the ability to repress a reporter gene in *E. coli*, which is a prominent property facilitating the construction of the expression vectors carrying a target gene. The β-galactosidase (*bgaB* gene) basal levels expressed from P*grac*01-*bgaB* could be repressed at least twice in the *E. coli* cloning strain. Second, the inducer-free production of BgaB from four different plasmids with the P*grac*01 promoter in *B. subtilis* was investigated. As expected, BgaB expression levels of inducer-free constructs are at least 37 times higher than that of the inducible constructs in the absence of IPTG, and comparable to those in the presence of the inducer. Third, using efficient IPTG-inducible expression vectors containing the strong promoter P*grac*100, we could convert them into inducer-free expression plasmids. The BgaB production levels from the inducer-free plasmid in the absence of the inducer were at least 4.5 times higher than that of the inducible vector using the same promoter. Finally, we used *gfp* as a reporter gene in combination with the two promoters P*grac*01 and P*grac*100 to test the new vector types. The GFP expression levels could be repressed at least 1.5 times for the P*grac*01-*gfp*+ inducer-free construct in *E. coli*. The inducer-free constructs P*grac*01-*gfp*+ and P*grac*100-*gfp*+ allowed GFP expression at high levels from 23 × 10^4^ to 32 × 10^4^ RFU units and 9–13% of total intracellular proteins. We could reconfirm the two major advantages of the new inducer-free expression plasmids: (1) Strong repression of the target gene expression in the *E. coli* cloning strain, and (2) production of the target protein at high levels in *B. subtilis* in the absence of the inducer.

**Conclusions:**

We propose a general strategy to generate inducer-free expression vector by using IPTG-inducible vectors, and more specifically we developed inducer-free expression plasmids using IPTG-inducible promoters in the absence of the LacI repressor. These plasmids could be an excellent choice for high-level production of recombinant proteins in *B. subtilis* without the addition of inducer and at the same time maintaining a low basal level of the recombinant proteins in *E. coli*. The repression of the recombinant gene expression would facilitate cloning of genes that potentially inhibit the growth of *E. coli* cloning strains. The inducer-free expression plasmids will be extended versions of the current available IPTG-inducible expression vectors for *B. subtilis*, in which all these vectors use the same cognate promoters. These inducer-free and previously developed IPTG-inducible expression plasmids will be a useful cassette to study gene expression at a small scale up to a larger scale up for the production of recombinant proteins.

## Background

The rod-shaped Gram-positive soil bacterium *Bacillus subtilis* is an attractive host for the production of recombinant proteins of biotechnological interests. Because of its easy handling, including the development of novel expression systems, high-cell-density growth and its classification as a generally recognized as safe (GRAS) organism based on the lack of pathogenicity and the complete absence of endotoxins.

The most important element of expression vectors is the promoter. Three types of promoters have been developed for the expression of recombinant genes: (i) constitutive, (ii) autoinducible, and (iii) inducible ones [1]. To generate expression vectors either new promoters can be isolated from bacterial genomes and tested or existing promoters can be engineered to enhance their strength. One example is the widely used promoter P*spac* [2], the first IPTG-inducible promoter for *B. subtilis* that consist of *E. coli lacO* operator and an early promoter of the *B. subtilis* phage SPO-1 in combination with the regulatory element, *E. coli* LacI repressor. To improve the IPTG-inducible expression vector, the P*grac* promoter [3], a derivative of the *groESL* promoter of *B. subtilis* was generated. By optimizing its UP element and the −35, −16, −10 and +1 regions, recombinant proteins accumulated up to about 30% of the total cellular proteins [4]. Similar modifications have been carried out with the core region of the *aprE* promoter of *B. subtilis* [5] and the *cry3Aa* [6] promoter, resulting in improved transcription activities in both cases.

Auto-inducible and constitutive expression vectors belong to the category of inducer-free expression vectors that harbor auto-inducible or constitutive promoters. One auto-inducible expression vector is based on the *srfA* promoter which displayed a cell-density dependent expression pattern. While the recombinant protein was present at a low level at the early exponential growth phase, it was highly expressed at the late exponential as well in the stationary phase [7]. The productivity of the P*srfA* promoter could be further enhanced by promoter engineering and by using the spore mutant strain BSG1682. This strain carries a deletion of the gene coding for sigma F [8].

IPTG-inducible promoters are widely used and well characterized to study gene expression in *B. subtilis*. However, using the same promoters for the construction of different types of vectors for inducible and inducer-free expression has not been reported so far. In this work, we converted IPTG-inducible into inducer-free expression vectors and experimentally proved their two important properties: (1) low basal level of expression in *E. coli* due to efficient repression, and (2) production of recombinant proteins at high levels in the absence of the inducer.

## Results and discussion

### Construction of the inducer-free expression vectors and control of basal levels in *E. coli*

**Fig. 1:**
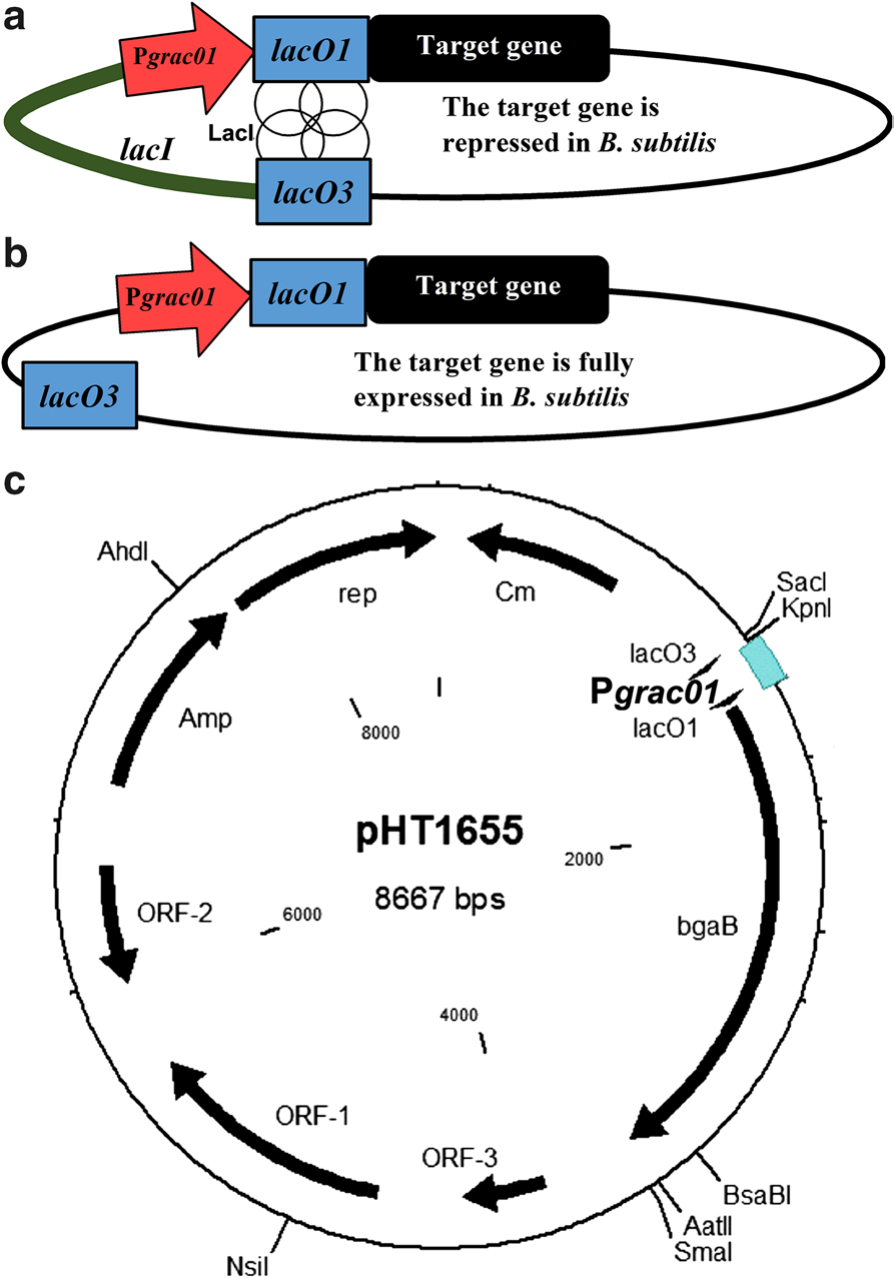
Conversion of an IPTG-inducible into an inducer-free expression vector for *B. subtilis*. **a** Schematic representation of the location of two *lacO* operators, e.g., in pHT01-*bgaB* and repression of the target gene by the presence of LacI (*circles*) encoded by *lacI* gene. **b** Removal of the *lacI* gene resulted in the inducer-free expression plasmid, such as pHT1655. **c** Map of the pHT1655 vector

The pHT01 expression vector and its derivatives contain the IPTG-inducible P*grac* (now P*grac*01) promoter [9]. Here, the promoter is flanked by two *lacO* operators, where one operator (*lacO1*) is located immediately downstream of the promoter and the second (*lacO3*) near the end of the *lacI* gene (Fig. 1a). Binding of the LacI repressor to both operators increases repression as shown elsewhere [10]. To convert this IPTG-inducible into an inducer-free expression vector for *B. subtilis*, we partially or fully deleted the *lacI* gene and preserving *lacO3* as shown diagrammatically (Fig. 1b). As an example, the inducer-free expression plasmid pHT1655 was constructed (Fig. 1c).

**Fig. 2:**
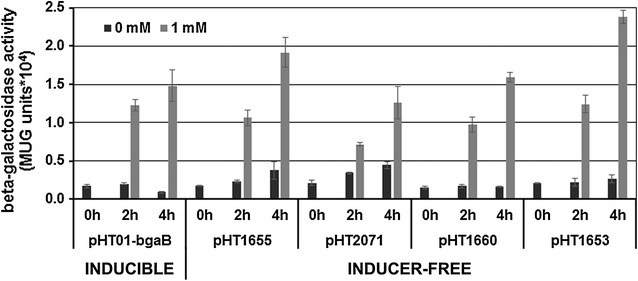
BgaB activities of five plasmids with the P*grac01* promoter in *E. coli*. The bacterial cells were grown in LB medium at 37 °C to the mid-logarithmic growth phase. Then, the culture was split into two subcultures and was further incubated in the absence of IPTG (0 mM) and the others induced with 1 mM IPTG. Samples were taken immediately after addition of IPTG (0 h) and 2 and 4 h later (2, 4 h). The β-galactosidase activity was measured in all samples and expressed as MUG units ×10^4^

Most of the expression vectors for *B. subtilis* are shuttle vectors which replicate both in *E. coli* and in *B. subtilis*. The cloning steps are carried out in *E. coli*, and the final expression vector containing the recombinant gene is then transformed into *B. subtilis* followed by its expression of the recombinant gene. In general, efficient expression vectors with strong promoters for *B. subtilis* also allow high protein production in *E. coli*. If constitutive or auto-inducible promoters are used, the basal level of expression in *E. coli* can create a problem depending on the expression level and the type of recombinant protein. Therefore, we constructed new expression vectors allowing repression in *E. coli* cloning strains. For the cloning purpose, the *E. coli* strain OmniMAX carries the *lacI* ^q^ allele on an F′ factor and should be able to repress expression from the pHT vectors carrying the P*grac01* promoter flanked by the two *lac* operators, *lacO1* and *lacO3* (see Fig. 1b). To test this assumption, plasmids pHT01-*bgaB* (control), and four inducer-free expression vectors containing P*grac01* promoter, pHT1655, pHT2071, pHT1660 and pHT1663 were transformed into *E. coli* OmniMAX. Cells of the five strains were grown in LB medium till the mid-log phase. Then, either no or 1 mM IPTG was added, and samples were withdrawn at t = 0 (immediately before addition of IPTG) and 2 and 4 h later. We used the *bgaB* gene coding for a heat-stable β-galactosidase (BgaB) as reporter gene [11]. The BgaB activities were determined in all samples, and the results are presented in Fig. 2. While the basal level of expression in the absence of the inducer IPTG remained low and comparable with all five plasmids analyzed, it could be induced after addition of IPTG from 1.3 × 10^4^ to 2.4 × 10^4^ 4 h after induction depending on the plasmid type (Fig. 2). The BgaB expression levels could be repressed at least 6.0-, 4.5-, 2.0-, 5.6- and 5.5-folds for pHT10-*bgaB*, pHT1655, pHT2071, pHT1660 and pHT1653, respectively. Among them, the basal level remained low in the absence of IPTG for plasmid pHT1660. Based on these data, plasmid pHT1660 should be used as a vector plasmid for cloning of genes whose products could harm the *E. coli* cells. In summary, the new expression vectors were able to repress target gene expression at least twice in *E. coli*, which might facilitate the cloning steps to generate stable recombinant vectors. Next, the new appropriate recombinant vectors were transformed into *B. subtilis*.

### Inducer-free production of BgaB from plasmids with the P*grac*01 promoter in *B. subtilis*

**Fig. 3:**
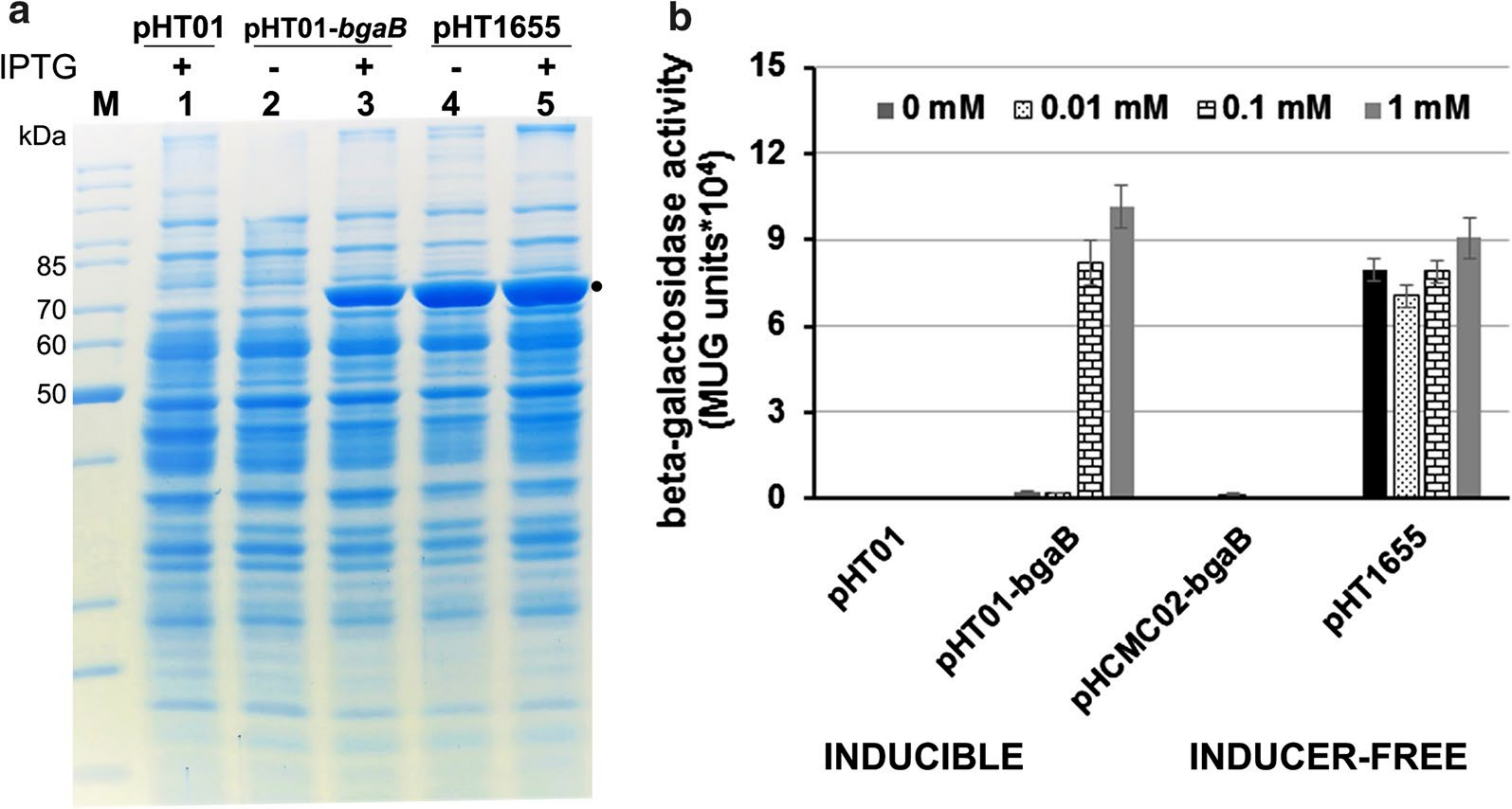
Expression of the *bgaB* reporter gene from inducible and inducer-free plasmids in *B. subtilis* 1012. Synthesis of the reporter protein BgaB expressed from the three different vectors pHT01-*bgaB* (P*grac*01-*bgaB*, inducible), pHCMC02-*bgaB* (P*lepA*-*bgaB*, inducer-free) [12] and pHT1655 (P*grac*01-*bgaB*, inducer-free) in the presence and absence of 1 mM IPTG. The empty vector pHT01 served as a negative control. The bacterial cells carrying these vectors were grown in LB medium at 37 °C to the mid-logarithmic growth phase. Then, the culture was split into four subcultures, where one was further incubated in the absence of IPTG (0 mM) and other three induced with 0.01, 0.1 and 1 mM IPTG. Samples were taken 2 h after addition of IPTG. **a** SDS-PAGE analysis of *bgaB* inducer-free expression in *B. subtilis* in the absence or presence of 1 mM IPTG and visualized by Coomassie brilliant blue staining. **b** The β-galactosidase activity was measured in all samples and expressed as MUG units (×10^4^)

The four plasmids pHT01 (P*grac*01, no *bgaB* gene), pHT01-*bgaB* (P*grac*01-*bgaB*, inducible), pHCMC02-*bgaB* (P*lepA*-*bgaB*, inducer-free) and pHT1655 (inducer-free) were transformed into the *B. subtilis* strain 1012. The strains were grown in LB medium in the presence of chloramphenicol to the mid-log phase. Then, IPTG was added at 0 (control), 0.01, 0.1 and 1.0 mM to each culture. Aliquots were collected 2 h later, and the BgaB activity or SDS-PAGE analysis were carried out as described in Materials and methods. First, using SDS-PAGE, we analyzed with the amount of BgaB produced by *B. subtilis* harboring plasmids carrying the P*grac*01 promoter: the empty vector pHT01, the IPTG-inducible vector pHT01-*bgaB*, and the inducer-free expression vector pHT1655. While no BgaB protein was synthesized with pHT01 as to be expected (Fig. 3a), it was IPTG-inducible in the presence of pHT01-*bgaB*, and synthesized in the presence and absence of IPTG with plasmid pHT1655 (Fig. 3a). Quantification of the BgaB protein bands using the ImageJ program revealed that BgaB accumulated up to 14% of cellular proteins for pHT1655 and equal to that of pHT01-*bgaB* in the presence of IPTG. In the case of the activities, the *B. subtilis* strain carrying pHT01 produced a very low level of β-galactosidase activities. While the amount of enzyme was also negligible for pHT01-*bgaB* in the absence of IPTG, it increased to 8 to 10 **×** 10^4^ units in the presence of 0.1 and 1.0 mM of IPTG (Fig. 3b). In contrast, cells harboring the pHT1655 plasmid produced up to 9 **×** 10^4^ units both in the absence and presence of IPTG. These results demonstrate that the *B. subtilis* strain carrying the plasmid pHT1655 produced BgaB in the absence of IPTG were 37 times higher as compared to plasmid pHT01-*bgaB* and at levels comparable to that after 1 mM IPTG-induction. We also analyzed expression of the *bgaB* gene from the plasmid pHCMC02-*bgaB* [12] where the gene is under control of the constitutive promoter P*lepA* from *B. subtilis*. The BgaB production level of the P*lepA*-*bgaB* construct was only at 0.14 **×** 10^4^ units (Fig. 3b). In comparison with the inducer-free pHT1655 plasmid, the BgaB production levels was 50 times higher than that of pHCMC02-*bgaB*. We conclude that the newly constructed vector pHT1655 allows BgaB production at high levels in an inducer-free manner.

**Fig. 4:**
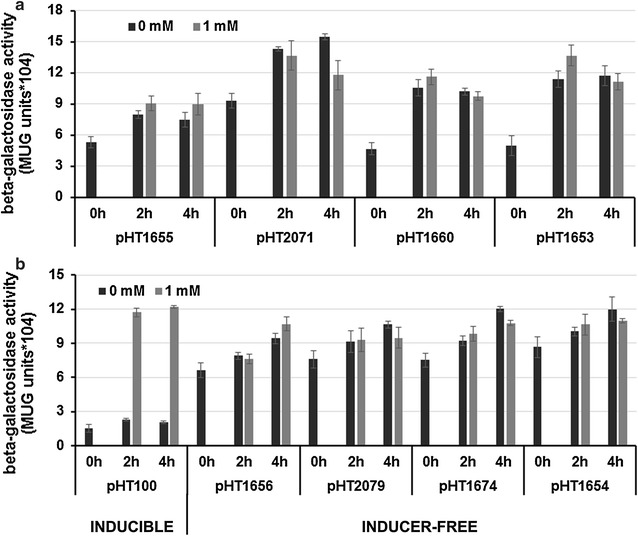
Influence of the spacer lengths between *lacO*1 and *lacO*3 on the β-galactosidase activity. The plasmids with different spacer lengths pHT1655 (143 bp), pHT2071 (280 bp), pHT1660 (548 bp) and pHT1653 (787 bp) carrying P*grac*01 (**a**) and pHT100 (inducible), pHT1656 (88 bp), pHT2079 (225 bp), pHT1674 (493 bp), pHT1654 (732 bp) harbouring P*grac*100 (**b**) were constructed. The deleted DNA region of *lacI* gene in the plasmids with P*grac*01 are identical to those with P*grac*100. The plasmids were transformed separately into *B. subtilis* 1012, and all strains were cultivated to an OD of 0.8. Then, the cultures were subdivided into two subcultures where IPTG was added at 1 mM to one of them. The BgaB activities were measured at time points 0 (immediately before addition of IPTG) and 2 and 4 h later

Next, we investigated whether the spacer length between the two *lacO* operators, *lacO*1 and *lacO*3, will influence the expression level. The operator *lacO*1 is located downstream of the promoter P*groES* and *lacO3* upstream of the promoter (Fig. 1a, b). Naturally, there is a *lacO3* the end of the *lacI* gene. By deletion of part of the *lacI* gene on the plasmids, we increased the spacer length from 143 bp (pHT1655) to 280 (pHT2071), 548 (pHT1660) and 787 bp (pHT1653), and measured the β-galactosidase activities as described. For all the inducer-free plasmids, BgaB activities in the absence of IPTG are in the same range with the inducible plasmid pHT01-*bgaB* in the presence of 1 mM IPTG (Figs. 3b, 4a). The 280 bp spacer (pHT2071) resulted in an increase from about 8 × 10^4^ in pHT1655 to 14 × 10^4^ units (Fig. 4a). This result would agree with the report that the different spacer lengths between *lacO*1 and *lacO*3 would enhance or reduce the target gene expression depending on the helix formation [13]. However, SDS-PAGE analysis of BgaB expression did not show significant differences between samples from four plasmids, and equal to that from pHT1655 (Fig. 3a). In summary, the results provided strong evidence that plasmids with the entire or partially deleted *lacI* gene could allow BgaB production at high levels in the absence of IPTG.

### Development of inducer-free expression vector using another IPTG-inducible plasmid with the strong promoter P*grac*100 for *B. subtilis*

So far, inducer-free plasmids have been successfully created based on the IPTG-inducible P*grac*01 promoter. However, many other IPTG-inducible promoters were created based on a genetic modification of the P*grac*01 promoter, and many resulting promoters conferred high recombinant protein production levels in *B. subtilis* [4, 14]. Among them, the P*grac*100 promoter was used to develop IPTG-inducible expression vectors allowing remarkably high protein production levels in *B. subtilis* and a relatively low basal expression level in *E. coli* [15]. Therefore, we next analyzed whether the vector with the strong synthetic P*grac*100 promoter could be converted into efficient inducer-free expression plasmids.

To answer this question, we constructed four additional P*grac*100-*bgaB* inducer-free vectors, named pHT1654, pHT1656, pHT1674 and pHT2079 and fused the *bgaB* reporter gene immediately downstream of the promoters. The spacer lengths between *lacO*1 and *lacO*3 are varied from 88 bp (pHT1656) to 225 bp (pHT2079), 493 bp (pHT1674) and 732 bp (pHT1654), in which the deleted DNA regions of *lacI* gene were identical to those in pHT1655 (P*grac*01-*bgaB*, 143 bp spacer), pHT2071 (P*grac*01-*bgaB*, 280 bp spacer), pHT1660 (P*grac*01-*bgaB*, 548 bp spacer), pHT1653 (P*grac*01-*bgaB*, 787 bp spacer), respectively. *B. subtilis* cells carrying these plasmids were then grown in LB medium to the mid-log phase, split into two subcultures where one remained untreated and the second was induced by addition of 1 mM IPTG. Samples were withdrawn immediately before adding the inducer IPTG and 2 and 4 h later. The β-galactosidase activities were measured in all samples and are presented in Fig. 4b. As can be seen from this figure, the BgaB activities expressed from the inducer-freed plasmids with P*grac*100 in the absence of IPTG are comparable to those from P*grac*01 and reached between 9 × 10^4^ and 12 × 10^4^ units after 4 h. These values are comparable to those obtained with plasmid pHT100 after 4 h after induction (Fig. 4b) and at least 4.5 times higher than that without induction. The expression levels of the P*grac*01-*bgaB* construct were comparable to P*grac*100-*bgaB* plasmids because these constructs contain strong promoters, and the activities of BgaB in the samples with high expression levels are not linear with the strengths of the promoters [16]. BgaB protein band analysis from SDS-PAGE using the imageJ programme showed that BgaB expression levels could reach around 25% of the total cellular proteins which correspond with the inducible vector pHT100 as reported [4]. We conclude that another IPTG-inducible expression vector with the strong promoter could be converted into an inducer-free expression plasmid.

### Application of inducer-free expression vectors to produce GFP in the cytoplasm of *B. subtilis*

In the last step, to check for the two major advantages of the inducer-free expression plasmids, we used another intracellular reporter protein, GFP, in combination with both promoters, P*grac*01 and P*grac*100. The two inducible expression vectors pHT10-*gfp*+ (P*grac*01-*gfp*+) and pHT1168 (P*grac*100-*gfp*+) were used as controls and four inducer-free expression plasmids, pHT1650 (P*grac*01-*gfp*+, 548 bp spacer), pHT1651 (P*grac*01-*gfp*+, 787 bp spacer, pHT1695 (P*grac*100-*gfp*+, 493 bp spacer) and pHT1696 (P*grac*100-*gfp*+, 732 bp spacer) were generated.

**Fig. 5:**
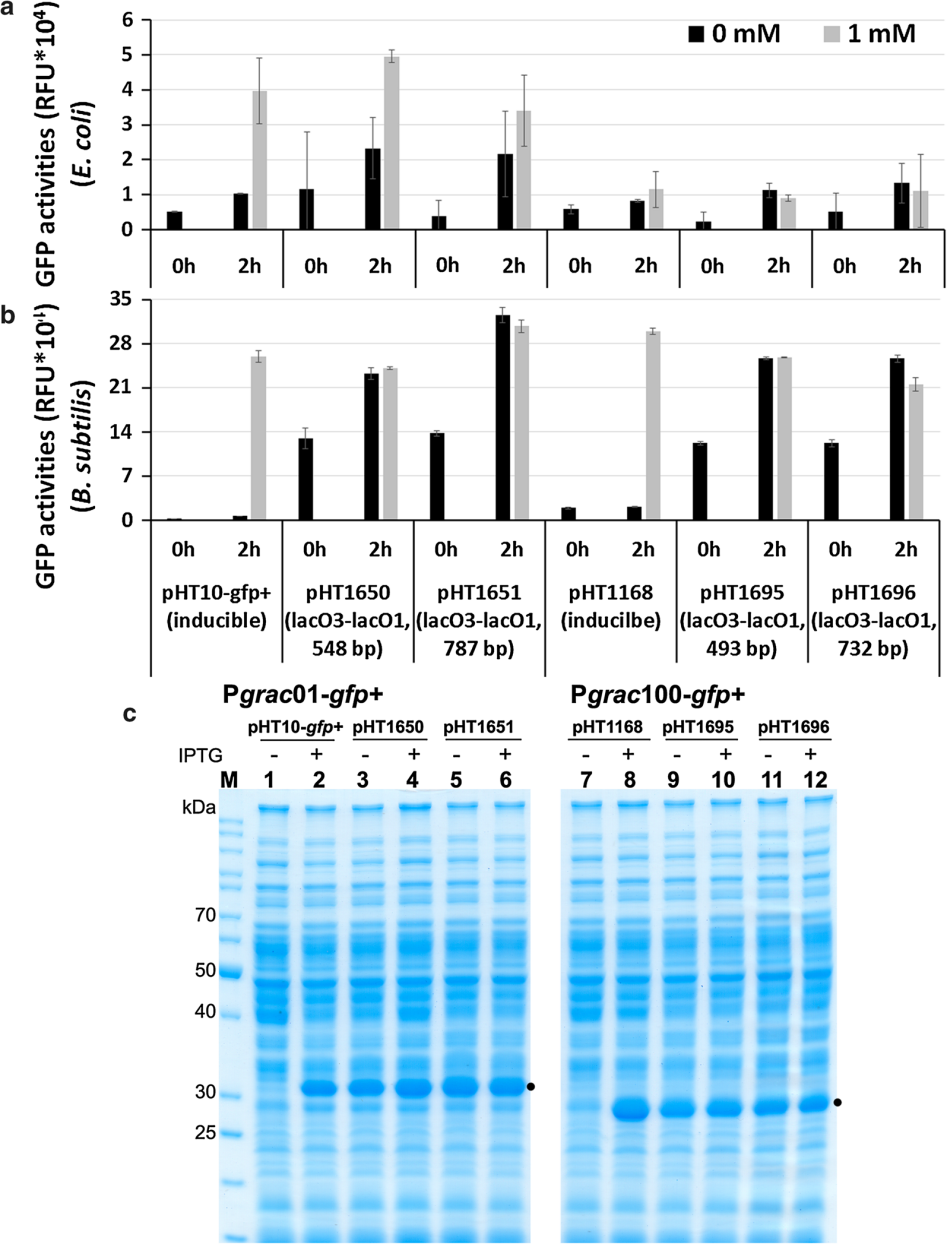
GFP expression using inducer-free plasmids based on P*grac*01 and P*grac*100. *E. coli* OmniMax and *B. subtilis* 1012 carrying expression plasmids with either the P*grac*01 promoter such as pHT10-*gfp*+ (inducible), pHT1650, pHT1651 or the P*grac*100 promoter such as pHT1168 (inducible), pHT1695, pHT1696 were generated. Cells were grown in LB medium to mid-log, and production of GFP was induced by addition of 1 mM IPTG. Aliquots were taken before and 4 h after addition of IPTG. Cells were lysed by lysozyme, and aliquots were measured for GFP activities and analyzed by SDS-PAGE. **a** Activities of *gfp* expression in *E. coli* to analyze for the repression ability of the inducer-free vector. **b** Activities of *gfp* expression in *B. subtilis* to measure the inducer-free production levels. **c** SDS-PAGE analysis of the *gfp* expression in *B. subtilis*; *black dots* indicate the positions of GFP

Next, we analyzed for the first advantage of the inducer-free vector that is the ability to repress the reporter gene expression in the *E. coli* cloning strain OmniMAX. Cells of the six strains were grown in LB medium till the mid-log phase. Then, either no or 1 mM IPTG was added, and samples were withdrawn at t = 0 (immediately before addition of IPTG) and 2 and 4 h later. The GFP fluorescence were measured in all samples, and the results are presented in Fig. 5a. The basal expression levels of the inducible vectors pHT10-*gfp*+ (P*grac*01-*gfp*+) and pHT1168 (P*grac*100-*gfp*+) ranking from 0.5 × 10^4^ to 1.2 × 10^4^ RFU were comparable to the P*grac*100-*gfp*+ inducer-free constructs, reaching 1.3 × 10^4^ RFU (Fig. 5a) after 2 h of incubation. The GFP expression levels could be repressed at least 1.5 times for the P*grac*01-*gfp*+ inducer-free construct. Also, when using the *E. coli* OmniMAX strain carrying plasmid pLacI (Novagen) that produces more LacI repressor, the reporter gene expression could be repressed at least 10 times (data not shown). Similar approaches using *E. coli* cloning strains with high levels of LacI expression could be used to reduce the basal levels, for example applying a *lacI* ^*Q1*^ strain [17]. These results confirmed that new inducer-free expression plasmids were able to retain at low basal levels or repress target gene expression in *E. coli*.

Then, we checked for the second advantage of the inducer-free vector, the ability allowing reporter gene expression in the absence of the inducer. We measured the GFP activities expressed in *B. subtilis* with the IPTG-inducible plasmids pHT10-*gfp*+ and pHT1168 and the four inducer-free plasmids pHT1650, pHT1651, pHT1695 and pHT1696. The GFP fluorescence could be induced about 100-fold with pHT10-*gfp*+ and about 15-fold with pHT1168 in *B. subtilis*. With the remaining four inducer-free plasmids, GFP activities accumulated from 23 × 10^4^ to 32 × 10^4^ RFU units in the 2 h samples and were comparable between the samples in the presence and absence of the inducer (Fig. 5b). The SDS-PAGE analysis also agreed with the previous data (Fig. 5c) and GFP production of about 9–13% of the total cellular proteins. The results reconfirmed that the newly developed inducer-free expression plasmid could allow GFP expression in the absence of inducer. They further reconfirmed two major benefits of the newly developed vectors: (i) to obtain a low basal level of recombinant proteins in *E. coli*, and (ii) to allow high protein production levels in *B. subtilis* in the absence of the inducer.

## Conclusions

This study proposed a general strategy to convert IPTG-inducible into inducer-free expression vectors. Specifically, we developed novel inducer-free expression plasmids containing IPTG-inducible promoters, P*grac*01 and P*grac*100, and checked for the production of the two reporter proteins BgaB and GFP. The novel inducer-free expression plasmids allowed high production levels of recombinant proteins in *B. subtilis* without the addition of inducer and at the same time maintained a low level of background expression or even repressed the recombinant gene expression in *E. coli*. The novel inducer-free expression plasmids are extended versions of the currently available IPTG-inducible expression vectors for *B. subtilis*, in which all these vectors use the same cognate promoters. This achievement will be a major milestone pushing forward the use of *B. subtilis* expression systems for over-production of recombinant proteins.

## Methods

### Bacterial strains, plasmids and growth conditions

*Escherichia coli* strain OmniMAX (Invitrogen) was used as a recipient in all cloning experiments and to determine expression levels. *B. subtilis* strain 1012 [18] was used to analyze expression of the two reporter genes *bgaB* and *gfp*+. A list of the plasmids and oligonucleotides used in this study is shown in Table 1. Cells were routinely grown in Luria broth (LB) at 37 °C under aeration and shaking at 200 rpm. Antibiotics were added where appropriate (ampicillin at 100 µg/mL for *E. coli* and chloramphenicol at 10 µg/mL for *B. subtilis*).

**Table 1:** Bacterial strains, plasmids and oligonucleotides used in this study

Bacterial strains	Genotype	Source/reference
*E. coli* OmniMAX	F′ {*pro*AB *lac*I^q^ *lac*ZΔM15 *Tn*10(Tet^R^) Δ(*ccdA*B)} *mcr*A Δ(*mrr hsd*RMS-*mcr*BC) Φ 80(*lac*Z)ΔM15 Δ(*lac*ZYA-*arg*F)U169 *end*A1 *rec*A1 *sup*E44 *thi*-1 *gyr*A96 *rel*A1 *ton*A *pan*D; used for cloning	Invitrogen
*B. subtilis* 1012	*leuA8 metB5 trpC2 hsrM1*	[18]

Plasmids	Description	Source/references
pHCMC02-*bgaB*	P*lepA*-*bgaB*, inducer-free expression plasmid	[12]
pNDH33-*bgaB*	P*grac01*-*bgaB, inducible*	[3]
pLacI	Expression of LacI, used with *E. coli* cloning strain	Novagen
pHT01	P*grac01,* inducible	[9]
pHT01-*bgaB*	P*grac01*-*bgaB,* inducible	[9]
pHT24	P*grac01,* Strep-tag II, inducible	[9]
pHT10-*gfp*+	P*grac01*-*gfp*+, inducible	[9]
pHT100	P*grac100*-*bgaB*, inducible	[4]
pHT255	P*grac100,* inducible	[4]
pHT1168	P*grac100*-*gfp*+, inducible	[4]
pHT1650	P*grac01*-*gfp*+*, ΔlacI, lacO1*-*lacO3* 548 bp	This study
pHT1651	P*grac01*-*gfp*+*, ΔlacI, lacO1*-*lacO3* 787 bp	This study
pHT1653	P*grac01*-*bgaB, ΔlacI, lacO1*-*lacO3* 787 bp	This study
pHT1654	P*grac100*-*bgaB, ΔlacI, lacO1*-*lacO3* 732 bp	This study
pHT1655	P*grac01*-*bgaB, ΔlacI, lacO1*-*lacO3* 143 bp	This study
pHT1656	P*grac100*-*bgaB, ΔlacI, lacO1*-*lacO3* 88 bp	This study
pHT1657	Basic plasmid, used to construct pHT1655	This study
pHT1658	Basic plasmid, used to construct pHT1656, pHT1681, pHT1682	This stuy
pHT1660	P*grac01-bgaB, ΔlacI, lacO1*-*lacO3* 548 bp	This study
pHT1661	Basic plasmid, used to construct pHT1660	This study
pHT1674	P*grac100*-*bgaB, ΔlacI, lacO1*-*lacO3* 493 bp	This study
pHT1695	P*grac100*-*gfp*+*, ΔlacI, lacO1*-*lacO3* 493 bp	This study
pHT1696	P*grac100-gfp*+*, ΔlacI, lacO1*-*lacO3* 732 bp	This study
pHT2071	P*grac01*-*bgaB, ΔlacI, lacO1*-*lacO3* 280 bp	This study
pHT2079	P*grac100*-*bgaB, ΔlacI, lacO1*-*lacO3* 255 bp	This study

Oligonucleotide	Sequence 5′→3′	Used for
ON224	CCGGATGACGTCGAATTCTAAACCTTCCCGGCTTCATCATG	pHT1655, pHT1660, pHT1656
ON925	GAATTAGCTTGGTACCAAAGGAGGTAAGGATCACTAG	To amplify promoter P*grac*100 to construct pHT1674
ON926B	GACGTCGACTCTAGACATGGATCCTTCCTCCTTTAATTGG	To amplify promoter P*grac*100 to construct pHT1674
ON941	AAAGGAGGAAGGATCCATGAATGTGTTATC	pHT1655, pHT1660, pHT1656
ON985	CAATTGCGTTGCGCTCACTGCCGGTAC	pHT1657, pHT1658
ON986	CGGCAGTGAGCGCAACGCAATTGAGCT	pHT1657
ON1975	CAATTGCGTTGCGCTCACTGCCAGCGCT	To create *lacO*3 in pHT2071
ON1976	AGCGCTGGCAGTGAGCGCAACGCAATTGAGCT	

P*grac*01 (another name is P*grac*) and P*grac*100 are the name of two different promoters

### Construction of expression vectors

#### Basic inducer-free expression vectors

To generate the first two basic inducer-free expression vectors, we removed the *lacI* and the *lacO3* sequences from plasmids pHT24 and pHT255, respectively, and inserted *lacO3* between the *Kpn*I and *Sac*I restriction sites by using the complementary oligonucleotides (ON) ON985 and ON986, resulting in the new plasmids pHT1657 and pHT1658. The third basic expression vector, pHT1661, was constructed by cutting pHT24 with *Sna*BI and *Eco*RV followed by Klenow enzyme treatment and religation with T4 DNA ligase to remove part of the *lacI* gene.

#### Inducer-free plasmids containing P*grac*01-*bgaB* and P*grac*01-*gfp*+

To construct pHT1655 and pHT1660, we amplified the *bgaB* gene using the primer pairs, ON224 and ON941 with pNDH33-*bgaB* as a template [3]. The *Bam*HI/*Aat*II-treated PCR product was introduced into pHT1657 and pHT1661 at the *Bam*HI and *Aat*II sites, respectively. To construct pHT1651 and pHT1653, we cut pHT01-*bgaB* [9] and pHT10-*gfp*+ with *Sna*BI/*Apa*I, treated the DNAs with Klenow enzyme and religated to remove part of the *lacI* gene. To construct plasmid pHT2071, we removed *lacI* together with the *lacO3* sequence from plasmid pHT01-*bgaB* and inserted the *lacO3* sequence between the *Sna*BI and *Sac*I restriction sites by using the two complementary oligonucleotides ON1975 and ON1976. To obtain pHT1650, we treated pHT10-*gfp*+ with *Sna*BI/*Eco*RV and followed by religation to remove part of the *lacI* gene.

#### Inducer-free P*grac*100-*bgaB* plasmids

To generate pHT1654 and pHT2079, we digested pHT100 *with Sna*BI/*Apa*I *or Sna*BI/*Sac*I, followed by treatment with Klenow enzyme and religation with T4 DNA ligase to remove major parts of the *lacI* gene. To construct pHT1656, we amplified the *bgaB* gene using the primer pairs ON224 and ON941 with pNDH33-*bgaB* as a template. The *Bam*HI/*Aat*II-treated PCR product was introduced into pHT1658 cut with *Bam*HI and *Aat*II. To construct pHT1674, we amplified the P*grac*100 promoter using the primer pair ON925 and ON926B and pHT100 as a template. The *Kpn*I/*Bam*HI-treated PCR product was ligated into pHT1660 cut with *Kpn*I and *Bam*HI. To construct pHT1695, we cut pHT1168 with *Sna*BI/*Eco*RV and religated to remove a major part of *lacI* gene. To construct pHT1696, we cut pHT1168 with *Sna*BI/*Apa*I, treated with Klenow and followed by religation to remove a major part of the *lacI* gene.

### Measurement of the BgaB and GFP production levels in *E. coli* and *B. subtilis*

Three single colonies of each strain were cultured in 5 mL LB liquid medium containing the appropriate antibiotic and shaken overnight at 200 rpm at room temperature (27 °C). A 1 mL pre-culture of each clone was transferred to 30 mL LB medium containing the appropriate antibiotic in 100 mL shake flasks and incubated at 37 °C at 200 rpm. When the OD_600_ of the culture reached 0.6–1, the cells were induced by addition of IPTG at the indicated concentrations. Aliquots of the cells were harvested before (0 h) and 2 or 4 h after addition of IPTG. The cells were collected in Eppendorf tubes at an OD_600_ of 2.4 after centrifugation. Samples were prepared for activity measurements and SDS-PAGE analyses. The cells were lysed by addition of lysozyme, and sample buffer was added to 150 µL and 8 µL each were applied to SDS-PAGE.

To prepare the samples for measurements of GFP and BgaB activities, the collected *E. coli* cells were resuspended in 300 μL PBS (140 mM NaCl, 2.7 mM KCl, 10 mM Na_2_HPO_4_·2H_2_O, 1.8 mM KH_2_PO_4_). Then, 12 μL of chloroform and 6 μL of 0.1% SDS were added followed by shaking for 1 h. *B. subtilis* cells were lysed in 300 μL PBS containing 1 mg/mL of lysozyme and incubated at 37 °C for 2 h. All samples were centrifuged at 10,000 rpm for 5 min and used for determination of the activities.

GFP fluorescence were measured by using a microplate fluorometer (Clariostar, BMG LabTech) and a 384-well plate (Black) with an excitation wavelength at 470 ± 8 nm and an emission wavelength at 515 ± 8 nm. Determination of the GFP expression was calculated as relative fluorescence unit (RFU) divided by the OD_600_ (dGFP/OD_600_). All data were averaged from three independent samples of each time point [7].

To quantify BgaB activities, 20 µL of the supernatant were added into each well of a 384-well plate (Black) containing 80 μL of Z-buffer (60 mM Na_2_HPO_4_·2H_2_O, 40 mM NaH_2_PO_4_·2H_2_O, 10 mM KCl, 1 mM MgSO_4_). 25 µL of 1 mg/mL 4-Methylumbelliferyl β-d-galactopyranoside (MUG) in dimethyl sulfoxide (DMSO) were added to each well, and the samples were incubated at 55 °C for 15 min. The reaction was stopped with 30 μL 1 M Na_2_CO_3_. The amount of fluorescence generated by β-gal-dependent MUG hydrolysis was quantified in a microplate fluorometer (Clariostar, BMG Labtech), using as a blank reference the assay with a cell-free culture medium sample. Arbitrary units of β-gal activity (MUG units) were calculated as follows: (Vl/Vs) × F_360/460_/(t × OD_600_); Vl, the volume of the lysis from the cell samples; Vs the volume of the samples used for the assay; F_360/460_, flourescence signals measure with an excitation wavelength at 360 ± 8 nm and an emission wavelength at 460 ± 8 nm: t, incubation time; OD_600_, OD of the collected samples [19].
